# Supplementary material for: Benchmarking of SpCas9 variants enables deeper base editor screens of BRCA1 and BCL2
Source: Nat Commun. 2022 Mar 14;13:1318. doi: 10.1038/s41467-022-28884-7 (PMC8921519; doi:10.1038/s41467-022-28884-7)
Supplement: Supplementary file 1 — Supplementary Information [file 41467_2022_28884_MOESM1_ESM.pdf]

Supplementary Information for Sangree, Griffith et al., Benchmarking of SpCas9 variants enables deeper base editor screens of *BRCA1* and *BCL2*

Supplementary Figures 1 – 8, provided as a compiled PDF.
Supplementary Data 1 – 8, provided as spreadsheets.
Source Data for Figures, provided as a spreadsheet.

SUPPLEMENTARY DATA

Supplementary Data 1: PAM-mapping counts, library annotation, replicate correlations. Associated with Figs 1,3.

Supplementary Data 2: HF-off-target counts, library annotation, replicate correlations. Associated with Fig 2.

Supplementary Data 3: variant off-target counts, library annotation, replicate correlations. Associated with Fig 4.

Supplementary Data 4: LFC and probability of being active calculations for all of the off-target datasets that are used to calculate the CFD scores. Associated with Figs 2,4.

Supplementary Data 5: *BRCA1* CBE data - WT, NG, SpG counts, WT library annotation (includes different controls), variant library annotation, replicate correlations. Associated with Fig 5.

Supplementary Data 6: *BRCA1* ABE data - WT, NG, SpG counts, WT library annotation (includes different controls), variant library annotation, replicate correlations. Associated with Fig 5.

Supplementary Data 7: *BCL2* data - NG-CBE and NG-ABE counts, library annotations, replicate correlations. Associated with Fig 7.

Supplementary Data 8: Primers and guide sequences used for validation experiments and the parameters used to run all validation samples in CRISPResso2. Associated with Figs 6,7.

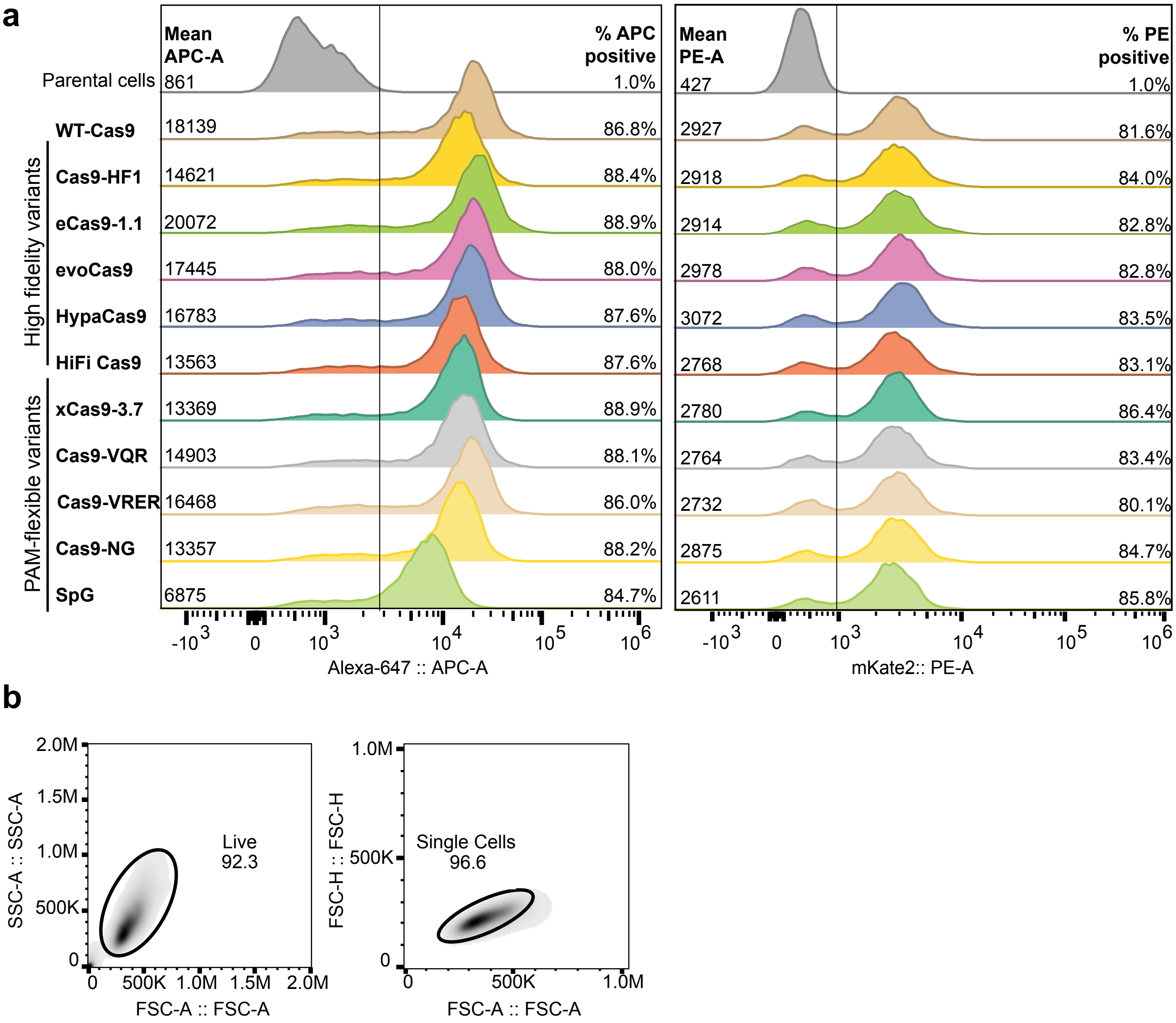

Supplementary Figure 1. Assessment of Cas9 protein expression levels. a) Ridge plots showing levels of Cas9 staining (left) and levels of mKate2 expression (right) for WT-Cas9 and all 10 variants assessed in this manuscript. This experiment was conducted after all screens had been performed with freshly prepared virus so that all Cas9 proteins could be assessed in parallel under the same conditions. b) Gating strategy used to assess fluorescence in the APC and PE channels. Stained parental A375 cells were gated first for live cells. This live cell population was then further gated to exclude doublets. Both gates were extrapolated to all Cas9 variants and the respective single cell populations were used to assess fluorescence intensity.

a

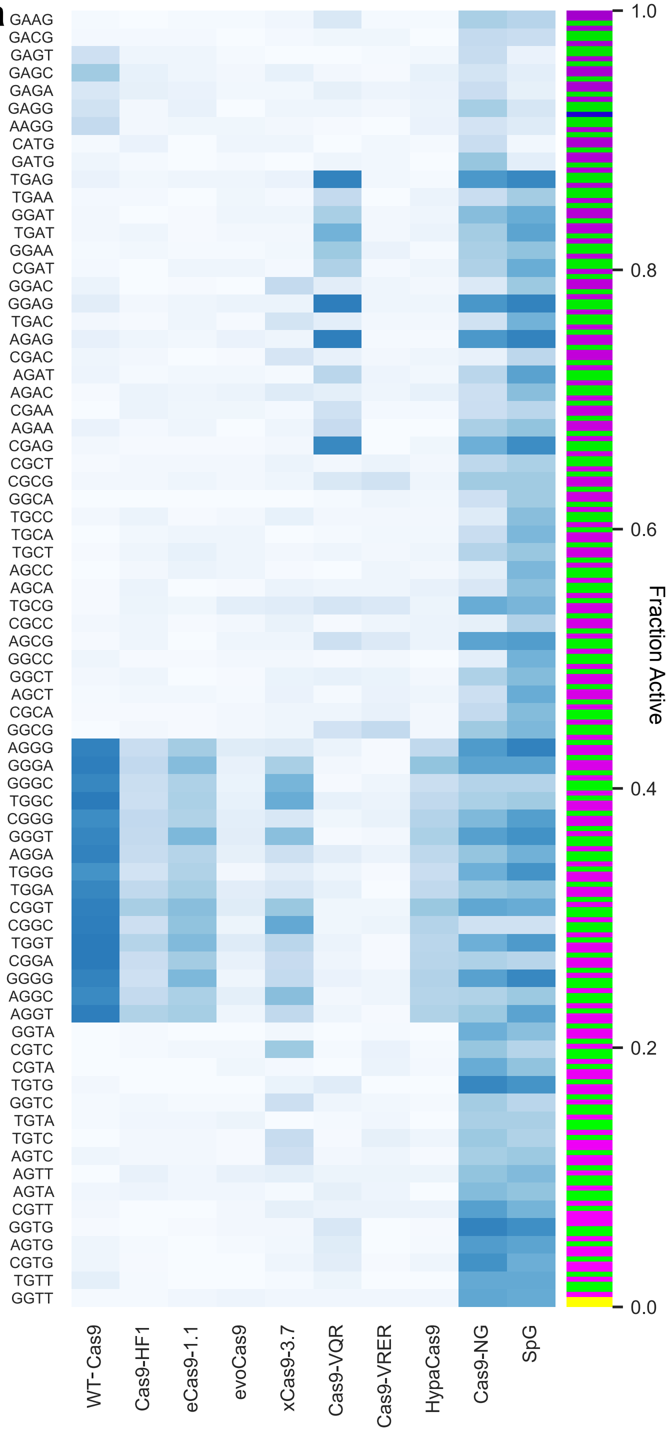

b

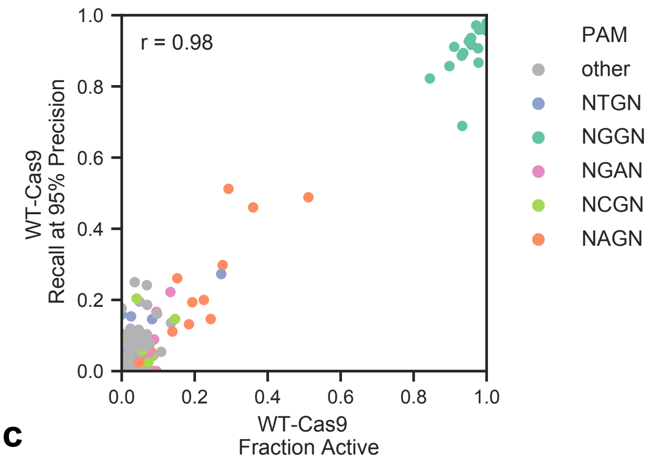

c

Cas	# Active g/G20	# Active G19	# Intermediate g/G20	# Intermediate G19
WT-Cas9	16	16	2	11
Cas9-HF1	0	14	13	7
evoCas9	0	0	0	20
HypaCas9	0	14	16	2
eCas9-1.1	0	15	16	7

Supplementary Figure 2: Establishment of a benchmarking assay for Cas9 activity. a) Heatmap of fraction active values for each variant. PAMs that have a fraction active of >= 0.3 with at least one variant are shown on the y-axis. b) Comparison of fraction active metric (x-axis) and recall at 95% precision metric (y-axis) applied to WT-Cas9. Each dot represents a 4 nucleotide PAM, shaded according to the legend on the right. c) Number of active and intermediate PAMs when considering G19 or g/G20 sgRNAs for WT-Cas9 and high-fidelity variants.

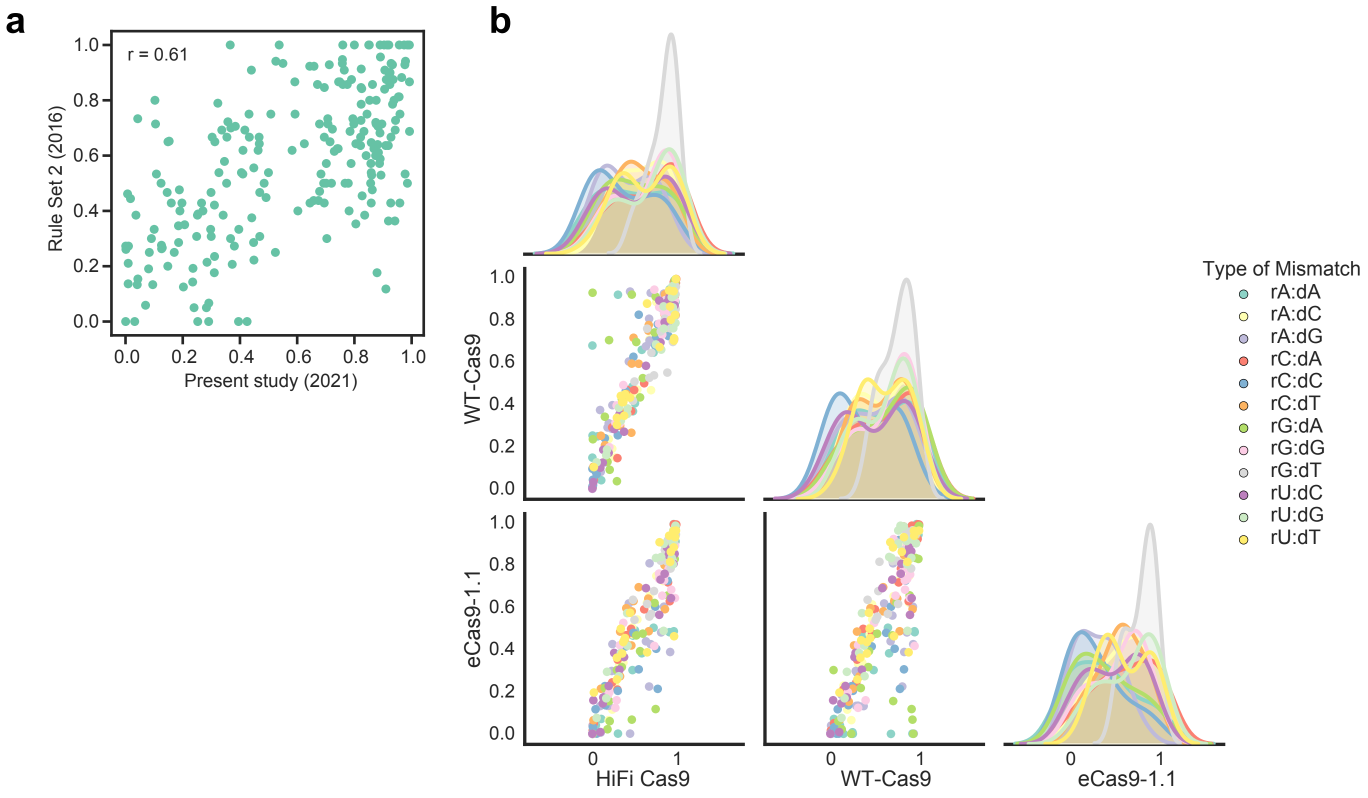

Supplementary Figure 3: Off-target profiles of high-fidelity variants. a) Comparison of CFD matrices for WT-Cas9 generated in this study (x-axis) and our previous work (y-axis). b) Comparison of the averaged probabilities of being active for each mismatch type between the Cas9 variants, shown as both scatter and kde plots.

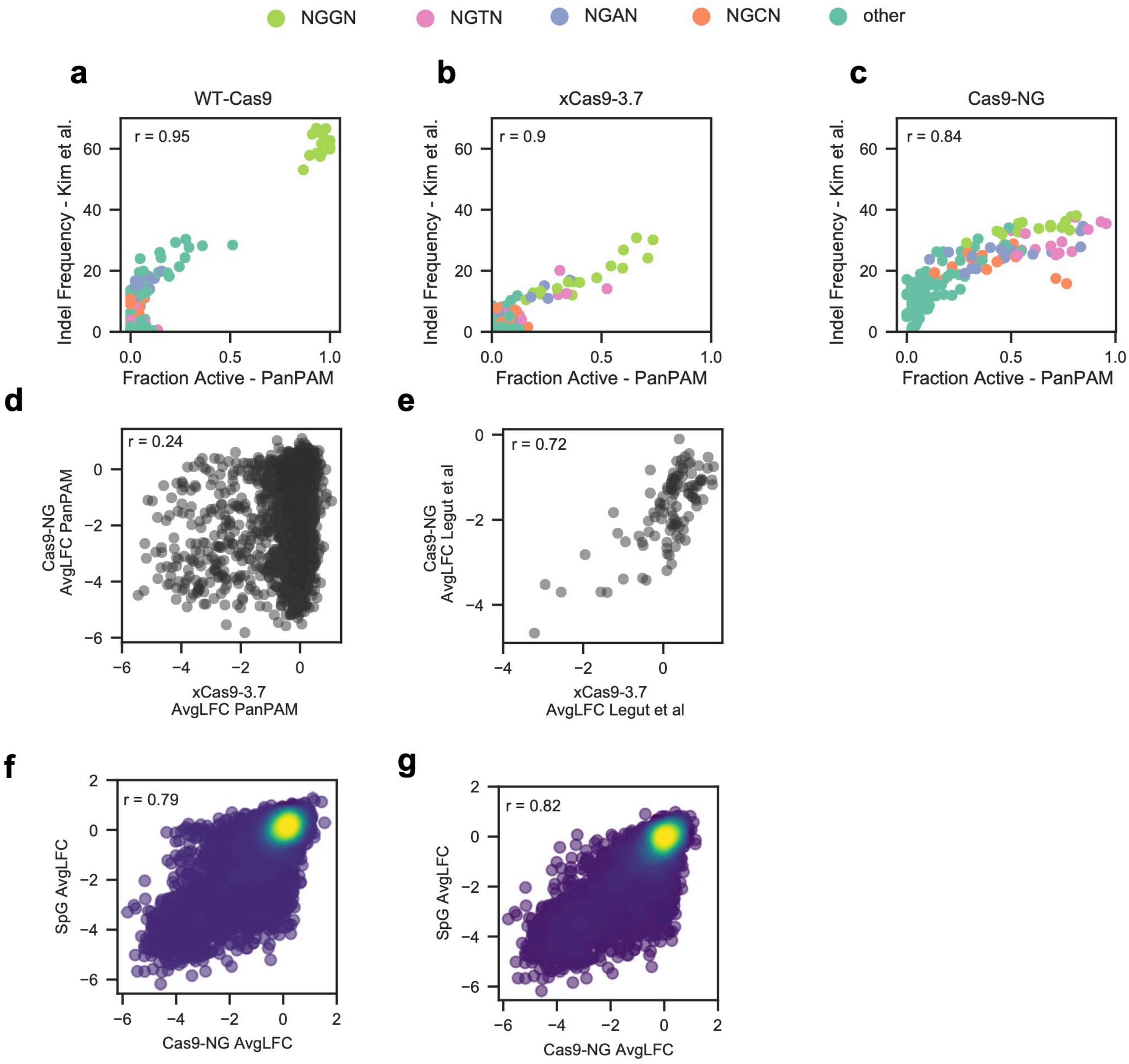

Supplementary Figure 4: Benchmarking PAM-flexible variants. a,b,c) Comparison of fraction active values for WT-Cas9, xCas9-3.7 and Cas9-NG from the present PanPAM study (x-axis) and indel frequencies from Kim et al. (y-axis). Each dot is a PAM. n = 148 PAMs. d) Comparison of avg LFC values for guides in the PAM-mapping library targeting essential genes with NG PAMs screened with xCas9-3.7 and Cas9-NG. n = 2747 sgRNAs. e) Comparison of avg LFC values for guides targeting CD45 and CD55 described in Legut et al. n = 108 sgRNAs. f) Comparison of sgRNAs screened with Cas9-NG and SpG. n = 18651 sgRNAs. g) Comparison of sgRNAs with an NG PAM screened with Cas9-NG and SpG. n = 4525 sgRNAs.

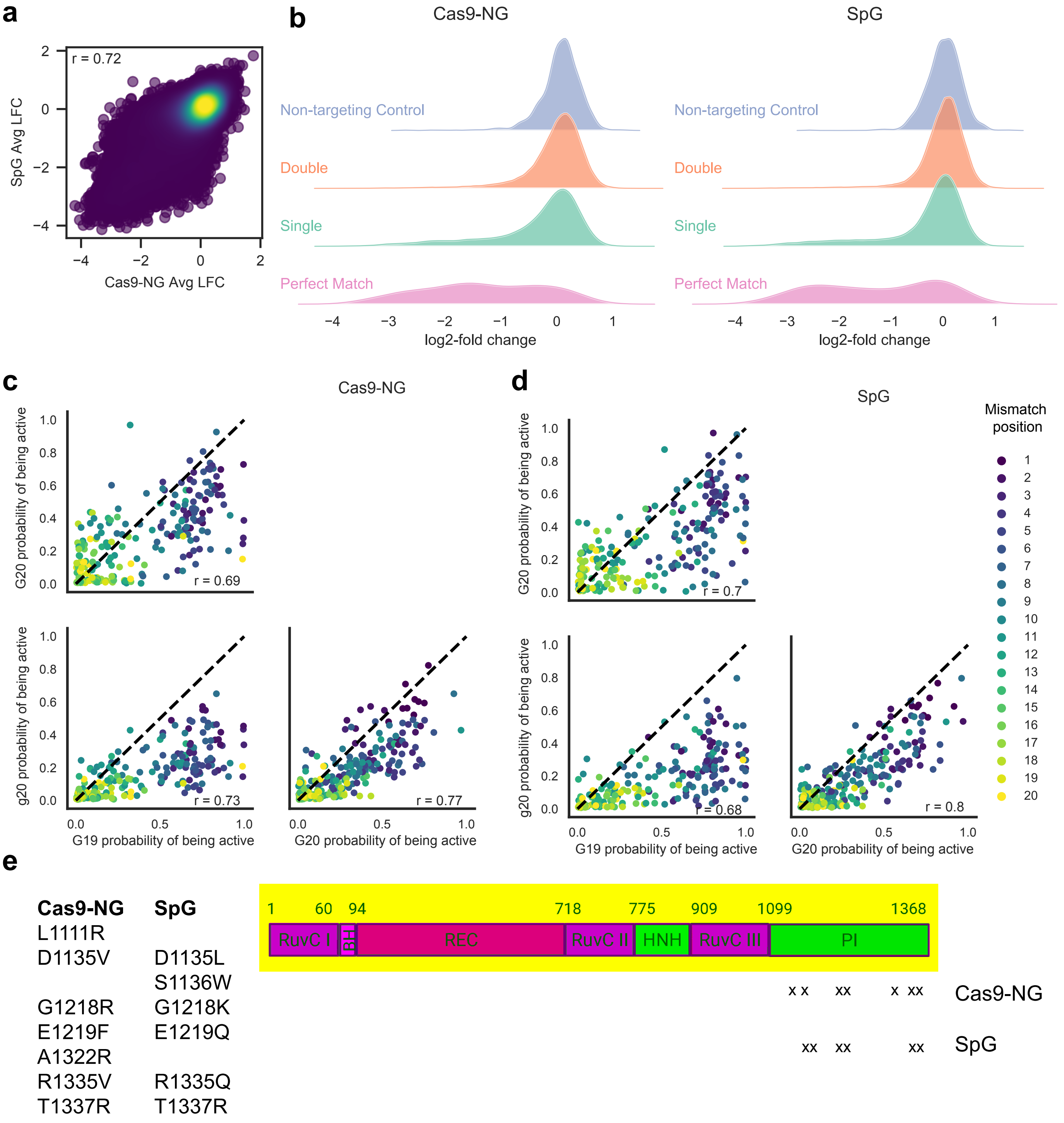

Supplementary Figure 5: Off-target profiles of Cas9-NG and SpG. a) Correlation between Cas9-NG and SpG, screened with the off-target library. Pearson's r is reported. n = 78058 sgRNAs. b) Ridge plots showing activity of unfiltered guides with zero, one or two mismatches. c) Scatter plots depicting the correlation between mismatches with sgRNAs of all 5'-types included in the library for Cas9-NG, colored by mismatch position. Each dot represents a type of mismatch at each position along the sgRNA (n = 228 pairings). d) Same as (c) but for SpG. e) Schematic depicting the mutations that result in Cas9-NG and SpG.

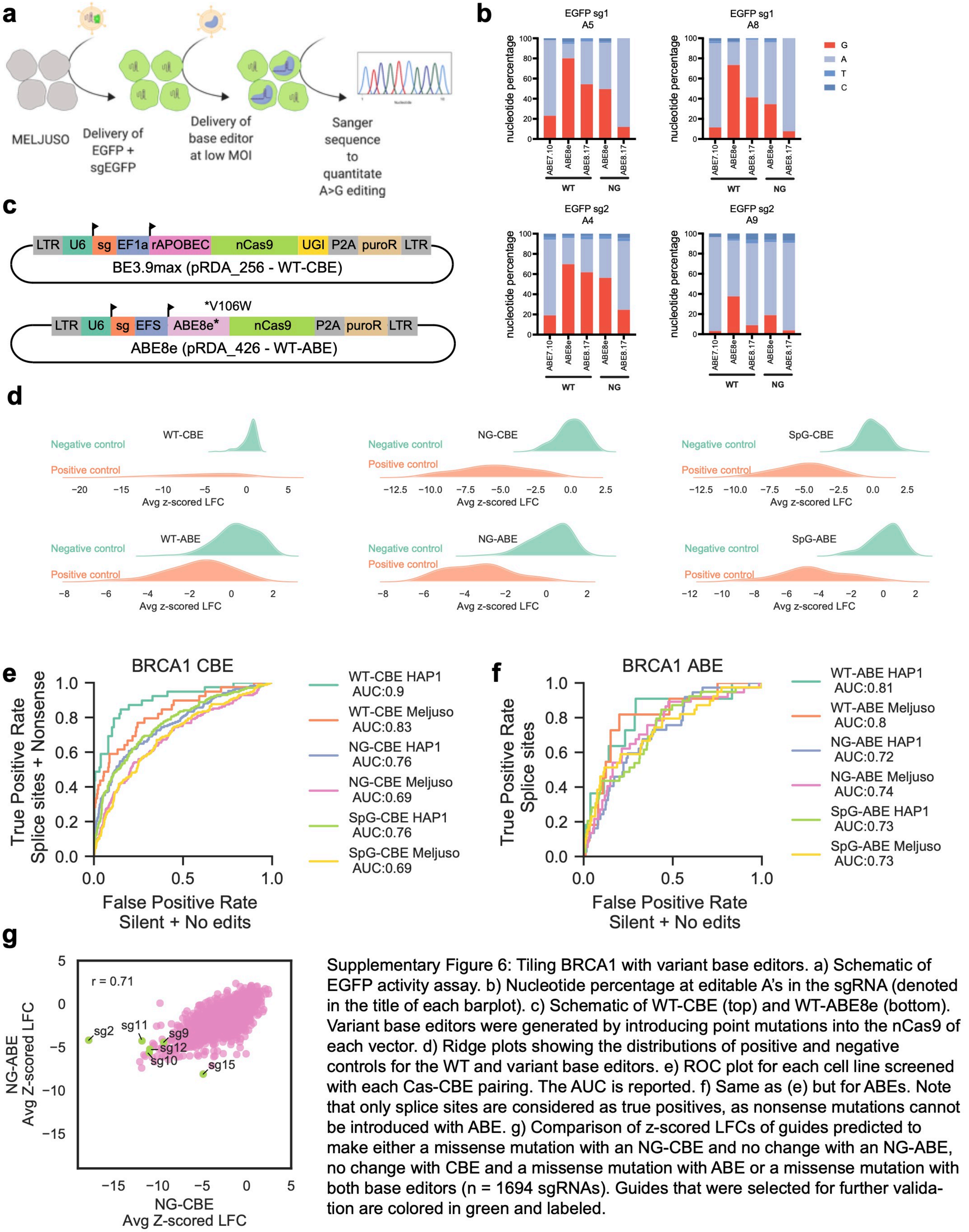

Supplementary Figure 6: Tiling BRCA1 with variant base editors. a) Schematic of EGFP activity assay. b) Nucleotide percentage at editable A's in the sgRNA (denoted in the title of each barplot). c) Schematic of WT-CBE (top) and WT-ABE8e (bottom). Variant base editors were generated by introducing point mutations into the nCas9 of each vector. d) Ridge plots showing the distributions of positive and negative controls for the WT and variant base editors. e) ROC plot for each cell line screened with each Cas-CBE pairing. The AUC is reported. f) Same as (e) but for ABEs. Note that only splice sites are considered as true positives, as nonsense mutations cannot be introduced with ABE. g) Comparison of z-scored LFCs of guides predicted to make either a missense mutation with an NG-CBE and no change with an NG-ABE, no change with CBE and a missense mutation with ABE or a missense mutation with both base editors (n = 1694 sgRNAs). Guides that were selected for further validation are colored in green and labeled.

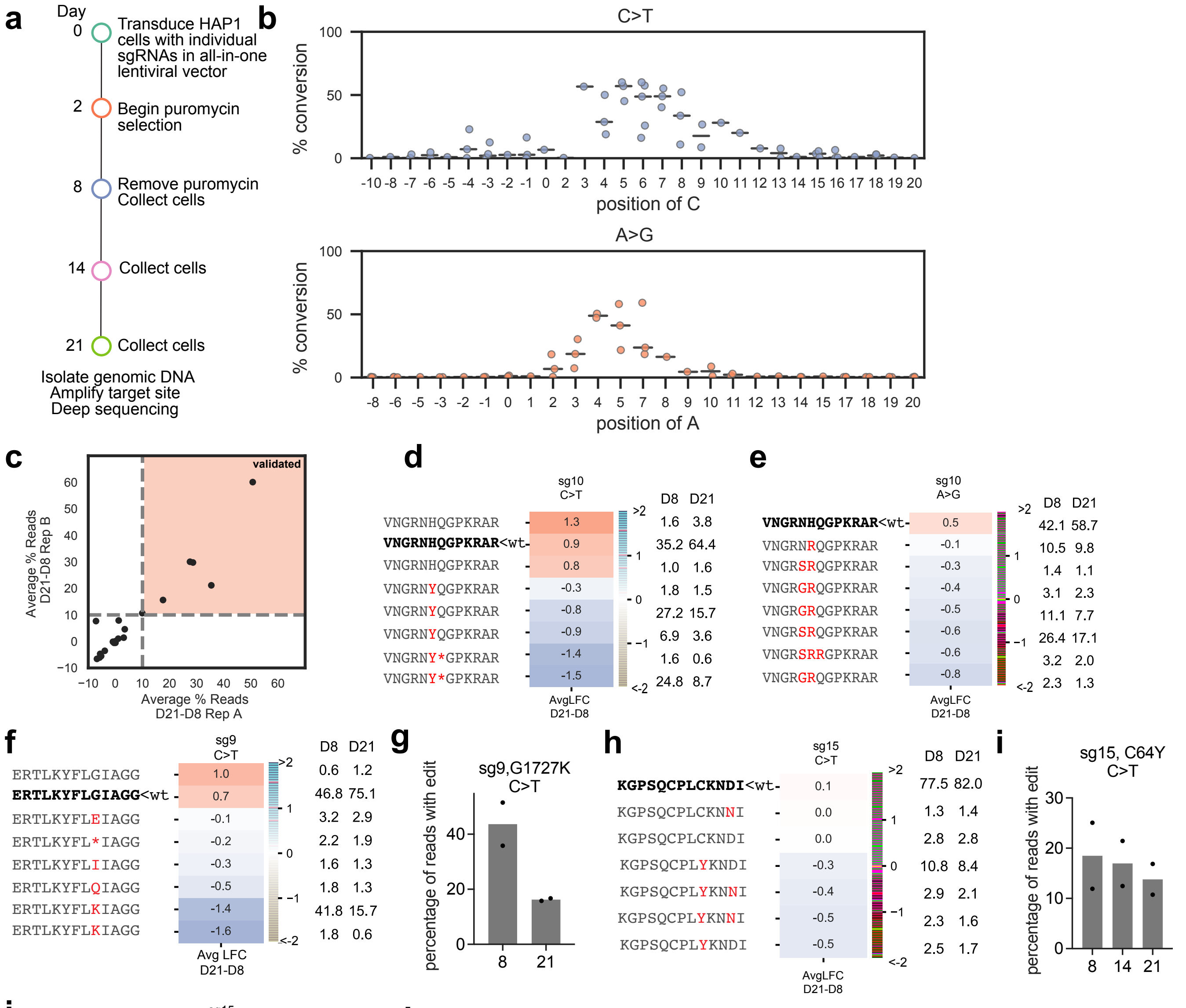

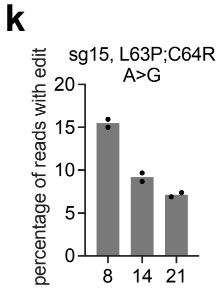

Supplementary Figure 7: Validation of BRCA1 hits identified by Cas9-NG. a) Timeline by which validation experiments were performed. b) C>T conversion (top, n=60) and A>G conversion (bottom, n=51) of guides with an intermediate/active PAM. Positions along the x-axis correspond with positions in the protospacer, where 1 corresponds to the first nucleotide of the protospacer and 21-23 correspond to the PAM. Lines (measure of center) show the median of all edits at that position across the validated sgRNAs. c) Validation scheme. sgRNAs in which the average % of reads increased by more than 10% from day 8 to day 21 are considered validated, as depicted with the orange shading. d,e,f,h,j) Translated sequence around the sgRNA for any allele with at least 1% abundance in any condition. The WT sequence is bolded in black, unchanged amino acids are in grey, and substitutions are highlighted in red. Avg LFC from day 21 - day 8 is indicated on the heatmap and relative percent abundance of each allele is indicated to the right (normalized after filtering for alleles with <1% abundance at both timepoints). g,i,k) Percentage of all sequencing reads containing the indicated mutation at each timepoint. Dots indicate n=2 biological replicates.

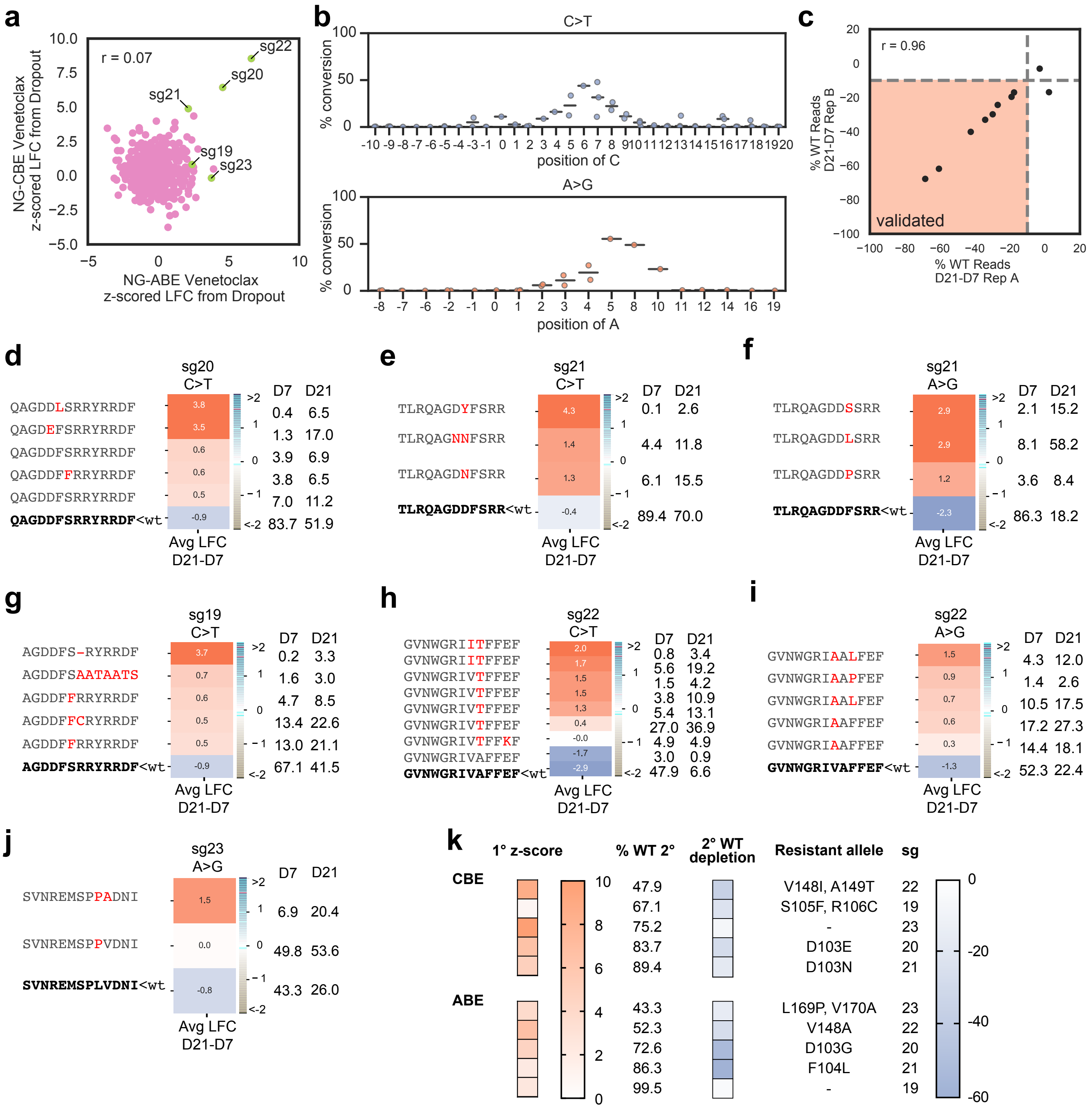

Supplementary Figure 8: Validation of BCL2 hits identified by Cas9-NG. a) Comparison of z-scored LFCs of guides predicted to make either a missense mutation with one base editor and a silent edit or no edit with the other, or a missense mutation with both base editors (n = 539 sgRNAs). Guides that were selected for further validation are colored in green and labeled. Pearson's r is reported. b) C>T conversion (top, n=63) and A>G conversion (bottom, n=26) with sgs 19-23. Positions along the x-axis correspond with positions in the protospacer, where 1 corresponds to the first nucleotide of the protospacer and 21-23 correspond to the PAM. Lines (measure of center) show the median of all edits at that position across sgs 19-23. c) Validation scheme. sgRNAs in which the average % of WT reads depletes by more than 10% (under selective pressure of Venetoclax) from day 7 to day 21 are considered validated, as depicted with the orange shading. d-j) Translated sequence around the sgRNA for any allele with at least 1% abundance in any condition. The WT sequence is bolded in black, unchanged amino acids are in grey, and substitutions are highlighted in red. Avg LFC from day 21 - day 7 is indicated on the heatmap and relative percent abundance of each allele is indicated to the right (normalized after filtering for alleles with <1% abundance at both timepoints). k) Summary of validation results. 1° z-score indicates the average z-scored LFC of the sgRNA in the primary screen. % WT 2° indicates the % of reads that were still WT (unedited) on day 7 in the validation experiment. 2° WT depletion indicates the average change in the abundance of the WT allele from day 7 to day 21 in the Venetoclax-treated arm of the validation experiment.
