# Supplementary material for: Benchmarking of SpCas9 variants enables deeper base editor screens of BRCA1 and BCL2
Source: Nat Commun. 2022 Mar 14;13:1318. doi: 10.1038/s41467-022-28884-7 (PMC8921519; doi:10.1038/s41467-022-28884-7)
Supplement: Supplementary file 2 — Reporting Summary [file 41467_2022_28884_MOESM2_ESM.pdf]

nature portfolio

Corresponding author(s): John Doench

Last updated by author(s): Feb 6, 2022

Reporting Summary

Nature Portfolio wishes to improve the reproducibility of the work that we publish. This form provides structure for consistency and transparency in reporting. For further information on Nature Portfolio policies, see our Editorial Policies and the Editorial Policy Checklist.

Statistics

For all statistical analyses, confirm that the following items are present in the figure legend, table legend, main text, or Methods section.

n/a	Confirmed	
☐	☒	The exact sample size (*n*) for each experimental group/condition, given as a discrete number and unit of measurement
☐	☒	A statement on whether measurements were taken from distinct samples or whether the same sample was measured repeatedly
☐	☒	The statistical test(s) used AND whether they are one- or two-sided<br>*Only common tests should be described solely by name; describe more complex techniques in the Methods section.*
☒	☐	A description of all covariates tested
☒	☐	A description of any assumptions or corrections, such as tests of normality and adjustment for multiple comparisons
☐	☒	A full description of the statistical parameters including central tendency (e.g. means) or other basic estimates (e.g. regression coefficient) AND variation (e.g. standard deviation) or associated estimates of uncertainty (e.g. confidence intervals)
☐	☒	For null hypothesis testing, the test statistic (e.g. *F*, *t*, *r*) with confidence intervals, effect sizes, degrees of freedom and *P* value noted<br>*Give P values as exact values whenever suitable.*
☒	☐	For Bayesian analysis, information on the choice of priors and Markov chain Monte Carlo settings
☒	☐	For hierarchical and complex designs, identification of the appropriate level for tests and full reporting of outcomes
☐	☒	Estimates of effect sizes (e.g. Cohen's *d*, Pearson's *r*), indicating how they were calculated

Our web collection on statistics for biologists contains articles on many of the points above.

Software and code

Policy information about availability of computer code

Data collection	PoolQ versions 2.2.0 and 3.3.2 were used to deconvolute screens. Both are openly available at https://portals.broadinstitute.org/gpp/public/software/poolq.
Data analysis	Screens were analyzed using custom code written in R version 3.5.1 or Python version 3 that is available on Github (https://github.com/gpp-rnd/cas9-variants-manuscript). Python version 3and PRISM GraphPad version 8 were used for visualization. EditR v1.0.8 (available at https://github.com/MoriarityLab/EditR) was used to quantify editing percentages in the base editor activity assays. FlowJo (version 10.8.1) was used to analyze flow cytometry data.

For manuscripts utilizing custom algorithms or software that are central to the research but not yet described in published literature, software must be made available to editors and reviewers. We strongly encourage code deposition in a community repository (e.g. GitHub). See the Nature Portfolio guidelines for submitting code & software for further information.

Data

Policy information about availability of data

All manuscripts must include a data availability statement. This statement should provide the following information, where applicable:

- Accession codes, unique identifiers, or web links for publicly available datasets
- A description of any restrictions on data availability
- For clinical datasets or third party data, please ensure that the statement adheres to our policy

Source data are provided with this paper as a Source Data file. The read counts for all screening data and subsequent analyses are provided as Supplementary Data. Fastq files are deposited with the Gene Expression Omnibus (GSE180351 [https://www-ncbi-nlm-nih-gov.ezproxy.u-pec.fr/geo/query/acc.cgi?acc=GSE180351]) and the Sequence Read Archive (PRJNA753064 [https://ddbj.nig.ac.jp/resource/bioproject/PRJNA753064]).

Guide sequences for tiling libraries were designed using sequence annotations from Ensembl (GRCg38). We used Ensembl's REST API (https://rest.ensembl.org/) to obtain the genomic locations of transcripts, transcript sequences, and protein sequences, and used these to annotate each sgRNA with its predicted edits. We used NCBI's ClinVar database (https://www.ncbi.nlm.nih.gov/clinvar/) to annotate the clinical significance of variants introduced in the base editing screens.

Field-specific reporting

Please select the one below that is the best fit for your research. If you are not sure, read the appropriate sections before making your selection.

[x] Life sciences   [ ] Behavioural & social sciences   [ ] Ecological, evolutionary & environmental sciences

For a reference copy of the document with all sections, see nature.com/documents/nr-reporting-summary-flat.pdf

Life sciences study design

All studies must disclose on these points even when the disclosure is negative.

Sample size	No sample sizes were determined. Sample sizes are based on available data. All screens were performed such that each guide was present in at least 500 cells.
Data exclusions	Guides targeting BRCA1 and BRCA2 were excluded from the PAM-mapping screen analysis downstream of the calculation of log2-fold-changes because BRCA1 and BRCA2 are not widely panlethal.
Replication	All screens were performed in at least biological duplicates, which is customary for studies of this type. We performed the BRCA screens in HAP1 first, followed by the screens in MELJUSO at a later time point. We observed good correspondence between these cell lines screened at different times, and between all replicates in all screens.
Randomization	No human or animal subjects were used in the experimental conditions and therefore randomization is not relevant to this work.
Blinding	No human or animal subjects were used in the experimental conditions, and therefore blinding is not relevant to this work.

Reporting for specific materials, systems and methods

We require information from authors about some types of materials, experimental systems and methods used in many studies. Here, indicate whether each material, system or method listed is relevant to your study. If you are not sure if a list item applies to your research, read the appropriate section before selecting a response.

Materials & experimental systems

n/a	Involved in the study	
[ ]	[x]	Antibodies
[ ]	[x]	Eukaryotic cell lines
[x]	[ ]	Palaeontology and archaeology
[x]	[ ]	Animals and other organisms
[x]	[ ]	Human research participants
[x]	[ ]	Clinical data
[x]	[ ]	Dual use research of concern

Methods

n/a	Involved in the study	
[x]	[ ]	ChIP-seq
[ ]	[x]	Flow cytometry
[x]	[ ]	MRI-based neuroimaging

Antibodies

Antibodies used	Cells were stained for Cas9 using a Cas9 Mouse mAB Alexa Fluor 647 conjugated antibody (Cell Signaling Technology, catalog no. 48796s). This antibody was diluted 1:100.
Validation	In addition to the validation provided by the vendor, this antibody was validated in the sense that it stained our positive control (WT-Cas9) for Cas9 expression.

Eukaryotic cell lines

Policy information about cell lines

Cell line source(s)	A375, MELJUSO, MOLM13 were acquired from the Cancer Cell Line Encyclopedia.<br>HEK293T - ATCC (CRL-3216)<br>HAP1 - Horizon Discovery (C631)
Authentication	Cell lines were authenticated by SNP profiling.
Mycoplasma contamination	Cell lines routinely tested negative for mycoplasma (~bimonthly).

Commonly misidentified lines (See ICLAC register)	None.

Flow Cytometry

Plots

Confirm that:

- [x] The axis labels state the marker and fluorochrome used (e.g. CD4-FITC).
- [x] The axis scales are clearly visible. Include numbers along axes only for bottom left plot of group (a 'group' is an analysis of identical markers).
- [x] All plots are contour plots with outliers or pseudocolor plots.
- [x] A numerical value for number of cells or percentage (with statistics) is provided.

Methodology

Sample preparation	A375 cells acquired from the Cancer Cell Line Encyclopedia were transduced with WT-Cas9 and 10 Cas9 variants via lentivirus. Post selection, cells were fixed and permeabilized using the Abcam Cell Fixation and Permeabilization Kit (Abcam, catalog no. ab185917). Cells were stained as per the kit's protocol using Cas9 Mouse mAB Alexa Fluor 647 Conjugate (Cell Signaling Technology, catalog no. 48796s), diluted 1:100. Cells were washed with PBS two additional times to remove residual antibody and were resuspended in flow buffer ((PBS, 2% FBS, 5μM EDTA).
Instrument	Beckman Coulter CytoFLEX S flow cytometer
Software	Flowjo 10.8.1
Cell population abundance	At least 5,000 cells were analyzed per sample.
Gating strategy	FSC/SSC was used to select for live cells. This population was further gated to exclude doublets (FSC-H/FSC-A). We set gates in the APC-A and PE-A channels at 1% for stained parental A375 cells to assess fluorescence levels for all samples.

- [x] Tick this box to confirm that a figure exemplifying the gating strategy is provided in the Supplementary Information.
