# Supplementary material for: Benchmarking of SpCas9 variants enables deeper base editor screens of BRCA1 and BCL2
Source: Nat Commun. 2022 Mar 14;13:1318. doi: 10.1038/s41467-022-28884-7 (PMC8921519; doi:10.1038/s41467-022-28884-7)
Supplement: Supplementary file 4 — Description of Additional Supplementary Files [file 41467_2022_28884_MOESM4_ESM.pdf]

Title: Supplementary Data 1:
Description: PAM-mapping counts, library annotation, replicate correlations. Associated with Figs 1,3.

Title: Supplementary Data 2:
Description: HF-off-target counts, library annotation, replicate correlations. Associated with Fig 2.

Title: Supplementary Data 3:
Description: variant off-target counts, library annotation, replicate correlations. Associated with Fig 4.

Title: Supplementary Data 4:
Description: LFC and probability of being active calculations for all of the off-target datasets that are used to calculate the CFD scores. Associated with Figs 2,4.

Title: Supplementary Data 5:
Description: BRCA1 CBE data - WT, NG, SpG counts, WT library annotation (includes different controls), variant library annotation, replicate correlations. Associated with Fig 5.

Title: Supplementary Data 6:
Description: BRCA1 ABE data - WT, NG, SpG counts, WT library annotation (includes different controls), variant library annotation, replicate correlations. Associated with Fig 5.

Title: Supplementary Data 7:
Description: BCL2 data - NG-CBE and NG-ABE counts, library annotations, replicate correlations. Associated with Fig 7.

Title: Supplementary Data 8:
Description: Primers and guide sequences used for validation experiments and the parameters used to run all validation samples in CRISPResso2. Associated with Figs 6,7.
